# Valorizing the applicability of waste rubber tiles loaded with bagasse fibers in different epoxy resin systems

**DOI:** 10.1038/s41598-025-23117-5

**Published:** 2025-11-04

**Authors:** Alaa E. Elsayed Eldesoky, Ahmed M. Khalil, Ahmed Abd Allah Haroun, Samir Kamel

**Affiliations:** 1 Polymers and Pigments Department, National Research Centre, Dokki, Giza, 12622 Egypt; 2 Photochemistry Department, National Research Centre, Dokki, Giza, 12622 Egypt; 3 Department of Chemistry, Faculty of Science, Ain Shams University, Cairo, 11566 Egypt; 4 Cellulose and Paper Department, National Research Centre, Dokki, Giza, 12622 Egypt

**Keywords:** Waste rubber, Bagasse, Epoxy resin, Low cost tiles, Sustainability, Environmental sciences, Materials science

## Abstract

The efficient and economic exploitation of rubber tiles reinforced with epoxy resin with high adhesion properties is not just a theoretical concept, but a practical solution to the issue of waste rubber. The influence of different weight percentages for waste bagasse (0, 5, 10 and 15 wt%) on the mechanical performance of rubbery epoxy tile is investigated. Three commercial epoxy resins, Kemapoxy 131, Kemapoxy 175, and Kemapoxy, were blended with the aforementioned weight% of bagasse fibers (BF) parallel to waste tire powder (WTP) as a binding material at fixed 15% epoxy content at different agro-waste ratios. 12 formulas were mixed, pressed and molded. Hardness, swelling rate, abrasion resistance, and compression tests, along with the prepared tiles’ morphology, were measured at 10 wt% bagasse. Kemapoxy cast epoxy resin was used to provide a promising result for the abrasion test, which was 15 mg. However, at 15 wt% bagasse using Kemapoxy 175 polyurethane epoxy solvent free, it showed 20 mg at the same revolution test. The hardness of the tiles reached up to 87 Shore A. The swelling ratio decreased significantly with increasing bagasse content, achieving a minimum of 18% compared to 28% in the unfilled samples. Kemapoxy cast epoxy resin (R3) exhibited the most promising abrasion resistance, with a weight loss of only 15 mg/1000 cycles. Conversely, Kemapoxy 175 at 15 wt% BF showed slightly higher weight loss of 20 mg/1000 cycles. The prepared rubber tiles using Kemapoxy 175 displayed the highest compression strength, reaching 50 kN at 0% BF and 47 kN at 15 wt% BF, surpassing the values achieved with the other resins. These results confirm that the incorporation of BF significantly improves hardness and compression strength, while maintaining acceptable swelling and abrasion resistance. The given data can facilitate the industrial design of rubber tiles with desirable properties upon employing such resins, making the research highly relevant in the field of materials science and environmental engineering.

## Introduction

Reclaimed rubber tiles using epoxy resin can offer sustainable solutions for various applications. Research has shown the potential of incorporating waste rubber from tires into epoxy mortars, resulting in lightweight composites with low thermal conductivity^1^. Additionally, the development of bio-based liquid monomers derived from vanillin structures has enabled the creation of dynamic thermosets that can be fully depolymerized and recycled, showcasing the circular approach to material reuse^2^. Furthermore, deactivated cement asbestos powder has been successfully compounded with epoxy resins for flooring applications, demonstrating the feasibility of using waste materials as fillers in epoxy-based products^3^. Moreover, rubber-modified epoxy composites reinforced with nanomagnetic iron oxide have been designed for structural applications, showcasing enhanced mechanical properties and lightweight characteristics suitable for industrial use^4^. These studies collectively highlight the possibility of creating recycled rubber tiles using epoxy resin with improved properties and sustainability.

Waste tire rubber can be effectively utilized in flexible tile production through various methods^5–8^. Among researches, it has been shown that incorporating rubber aggregates as a partial replacement for coarse aggregates in concrete tile production is feasible, with a limit of up to 10% replacement to maintain optimal properties^9^. Another approach involves the direct molding of rubber granules and powders from tire recycling, showcasing the potential for large-scale production of rubber tiles without binders^10^. Additionally, a method involving extrusion and composite molding of rubber and plastic allows for the creating of rubber and plastic tiles using waste materials, demonstrating a comprehensive recycling process for tile manufacturing^11^. These methods contribute to sustainable practices by reusing waste rubber tires and offer benefits such as enhanced durability, anti-skidding properties, and sound absorption in the resulting tiles^12^. Loading various fillers into virgin or waste rubber has successfully paved the way for the produced substrates to contribute as promising construction materials^13–17^. Studies have shown that incorporating waste tire rubber in clay ceramics used for tile production can save energy during sintering, making it an environmentally friendly alternative^18^. Additionally, the use of waste tire rubber in concrete structures has been explored, showing a reduction in mechanical properties but an increase in impact resistance when rubber replaces natural aggregates^19^. Furthermore, the tensile properties and dynamic behavior of elastic rubber layers in sports surfaces have been evaluated, indicating that recycled rubber can enhance shock absorption and vertical deformation in synthetic sports surfaces^20^. Overall, utilizing reclaimed waste tire rubber in tile production offers a sustainable solution that helps in waste management and enhances the properties of construction materials.

Waste tire rubber can be utilized for technical flooring tile production, offering benefits like cost-effectiveness and noise reduction, as demonstrated in the multilayer granular recycled rubber study. It was reported^21^ that the static and dynamic behavior of multilayer materials manufactured from granular recycled rubber applied to technical flooring was investigated. This procedure was carried out by varying their thickness, density and granulometry, as these parameters can assist in determining the performance of the formulated materials. Recycled rubber from automotive tires can be used for sound insulation applications. The acoustic properties of different fractions for recycled rubber granules and textile chopped materials were compared with compact rubber and textile panels. The authors concluded that bulk recycled rubber materials have very good values for their acoustic properties, which enables them to be used in several areas of industry^22^. The ability of a material to withstand mechanical action that gradually tends to remove material from its surface, such as rubbing, scraping, or erosion, is known as abrasion resistance. A product will withstand erosion brought on by scraping, scratching, and other forms of mechanical wear if it has abrasion resistance. As a result, the substance can maintain its shape and integrity. According to Muhr and Roberts (1992), abrasion is largely caused by the detachment of tiny particles connected to either a structural unit or localized stresses in the rubber^23^. When wearing becomes a problem, abrasion-resistant materials can be employed for moving and fixed elements. Small particles (1–5 mm) are removed during abrasion, leaving surface pits behind. Large particles (> 5 mm) are then removed^24^.

This work aims to employ WTP and BF as wastes with affordable trials to facilitate the industrial design of waste rubber tiles with desirable properties. In addition, we seek to investigate the influence of different weight percentages for waste bagasse (0, 5, 10 and 15 wt%) on the mechanical performance of rubbery epoxy tile. To conduct the experiments, three commercial epoxy resins, Kemapoxy 131, Kemapoxy 175, and Kemapoxy, were reinforced with the aforementioned weight% of bagasse and waste rubber as a binding material at a fixed 15 wt% epoxy content. An extensive examination was conducted to thoroughly investigate the effect of rubber and bagasse waste with different types of epoxy resins on the mechanical properties of tiles. To comprehensively assess the impact of epoxy type on the mechanical properties of the prepared tiles, compression or impact resistance, hardness test and abrasion resistance were carried out. These meticulously designed tests provided a profound understanding of the structural characteristics exhibited by the epoxy-rubber tile composites. Specifically, the compression properties of the rubber-epoxy tiles furnished valuable insights into their capacity to withstand considerable compression forces. The flexural properties of these rubber-epoxy tiles facilitated an all-encompassing comprehension of their resistance to damaging force. The compression properties provided valuable insights into the material’s ability to withstand compression forces. This feature is particularly interesting due to the increased resistance to crack propagation and good impact resistance, as well as damping properties, assisting in reducing the vibrational energy of a structure.

## Materials and methods

### Materials

The WTP was acquired from tires consumed locally in Cairo, Egypt. After being cleaned, the used tires were chopped into tiny pieces. These parts were diced and ground to create a powder that resembled WTP. Quena Paper Industry Company in Egypt supplied BF, which were ground into a fine powder. Bagasse is composed mainly of cellulose, hemicelluloses, lignin, and other minerals, and its percentages are 45–55, 20–25, 20 and 2%, respectively.

Three epoxy resins were carefully selected and purchased from CMB Egypt, each recommended for use in rubber tiles. The first resin was Kemapoxy 131 premix (coded R1), a polyurethane-based epoxy in gray color (base A) and polyisocyanate (base B). The second was Kemapoxy 175 (coded R2), a solvent-free polyurethane epoxy (base A) with a polyamine hardener (base B). The third resin was Kemapoxy (coded R3), a cast transparent epoxy resin (base A) with a polyamine hardener (base B). The curing time and temperature were expertly adjusted before mixing.

### Sieve analysis

The sieve analysis, a precise gradation test, is an important method for assessing the particle size distribution of granular material. Using this approach, we were able to determine the exact particle sizes of WTP and BF. For 100 g, the diameters were classified as given in Table 1, with the primary WTP particle size falling between 300 and 425 microns, and the primary BF particle size ranging between 425 and 600 microns.

**Table 1:** Particle size distribution of WTP and BF (for 100 g).

Micron	850	600	425	300	80	< 60
WTP (g)	0.5	10	35	44	05	4.6
BF (g)	19.30	30.00	20.50	13.00	12.80	4.01

Significant values are in bold.

### Formulation of tiles

WTP and BF were used as received without further treatment. They were blended in a mechanical blender at 250 rpm, followed by resin addition, and mixed for 5 min. Thereafter, they were placed into the mold and pressed mechanically at 170 °C for 20 min. They were then released and cooled to obtain the required tiles. The tiles were formulated (T1-T12), as shown in Table 2.

**Table 2:** Composition of the prepared tiles from WTP, BF and epoxy resins.

	0% BF			5% BF				10% BF				15% BF			
Code	R1	R2	R3	Code	R1	R2	R3	Code	R1	R2	R3	Code	R1	R2	R3
T1	√	--	--	T4	√	**--**	**--**	T7	√	--	--	T10	√	**--**	**--**
T2	--	√	--	T5	**--**	√	**--**	T8	--	√	--	T11	**--**	√	**--**
T3	--	--	√	T6	**--**	**--**	√	T9	--	--	√	T12	**--**	**--**	√

### Characterization of the tiles

#### Compression test

The compression test was carried out using a universal compression testing machine; Universal Automatic Tensile Testing Machines. To establish the relationship between the pressing force and deformation, the compression device with a capacity of 1000 kN, 1 HP, 440 V, 3 phase, AC supply, manual control and maximum speed 20 mm/ min was used comply with ISO 7500-1/ASTM E4. The samples were compressed radially between two parallel horizontal plates that moved together and had facial areas greater than the projected area of the tiles. The load is applied and increased gradually at a constant rate until the test specimen fails due to cracking or breaking. The experiment was repeated three times for each pellet at different pressures for different shapes, and the average value was taken to determine the maximum compression force and the maximum deformation at maximum load.

#### Abrasion test

The Taber Abraser (Abrader) is a dependable tool for conducting accelerated Taber abrasion resistance and wear tests on flat specimens using the acclaimed Taber method Taber Rotary Abraser 5155. Samples are mounted on a rotating turntable and subjected to the wearing action of two abrasive wheels at a specific pressure. Taber abrasion using wear index was used to elucidate the abrasion resistance of the rubber tile. The sample with a diameter of 110 mm was placed into the Taber abrasion tester (Taber Rotary Abraser 5155, USA) with 70 rpm rolling speed for 1000 rounds (three samples/ formula), according to ASTM C1353, ASTM C217.

#### Hardness test

A durometer is commonly used to gauge the hardness of rubbers and elastomers. In this investigation, the shore hardness of the prepared tiles was determined using a shore A durometer according to ASTM D2240. Five distinct sites for hardness measurements were selected on the rubber soling materials wear side, and calculating the mean and standard deviations.

#### Swelling test

A swelling test was carried out in toluene following ASTM D471-15.24. Cured rubber pieces of the dimension 10 × 10 mm were weighed using an electronic digital balance at the accuracy of 0.0001 g, soaked into 150 mm of toluene column for 24 h at room temperature, then dried and weighed. Each run was repeated three times and the average value is taken. The swelling ratio (Q) is calculated from the following equation:

$$\:\text{Q}\:\text{\%}=\:\frac{\text{W}2-\text{W}1}{\text{W}1}\:\text{X}\:100$$

where: Q is the swelling ratio and *W*_*1*_ and *W*_*2*_ are the specimen’s weights before and after soaking into toluene, respectively.

### Morphological characterization

To view the developed microstructure for each blend sample, the Cryo-fracture surfaces of the Specimens were sputter-coated with a thin layer of gold. Then, they were examined for morphological structure through a Quanta 200 F field emission scanning electron microscope (FE-SEM, FEI Co., USA) at an accelerated voltage of 5 kT.

### Thermogravimetric analysis (TGA)

Thermogravimetric analysis (TGA) was performed with Perkin Elmer analyzer equipment (USA) (Switzerland) under a nitrogen atmosphere with a temperature range of 50 to 900 °C and a heating rate of 10 °C/min.

## Results and discussion

**Fig. 1:**
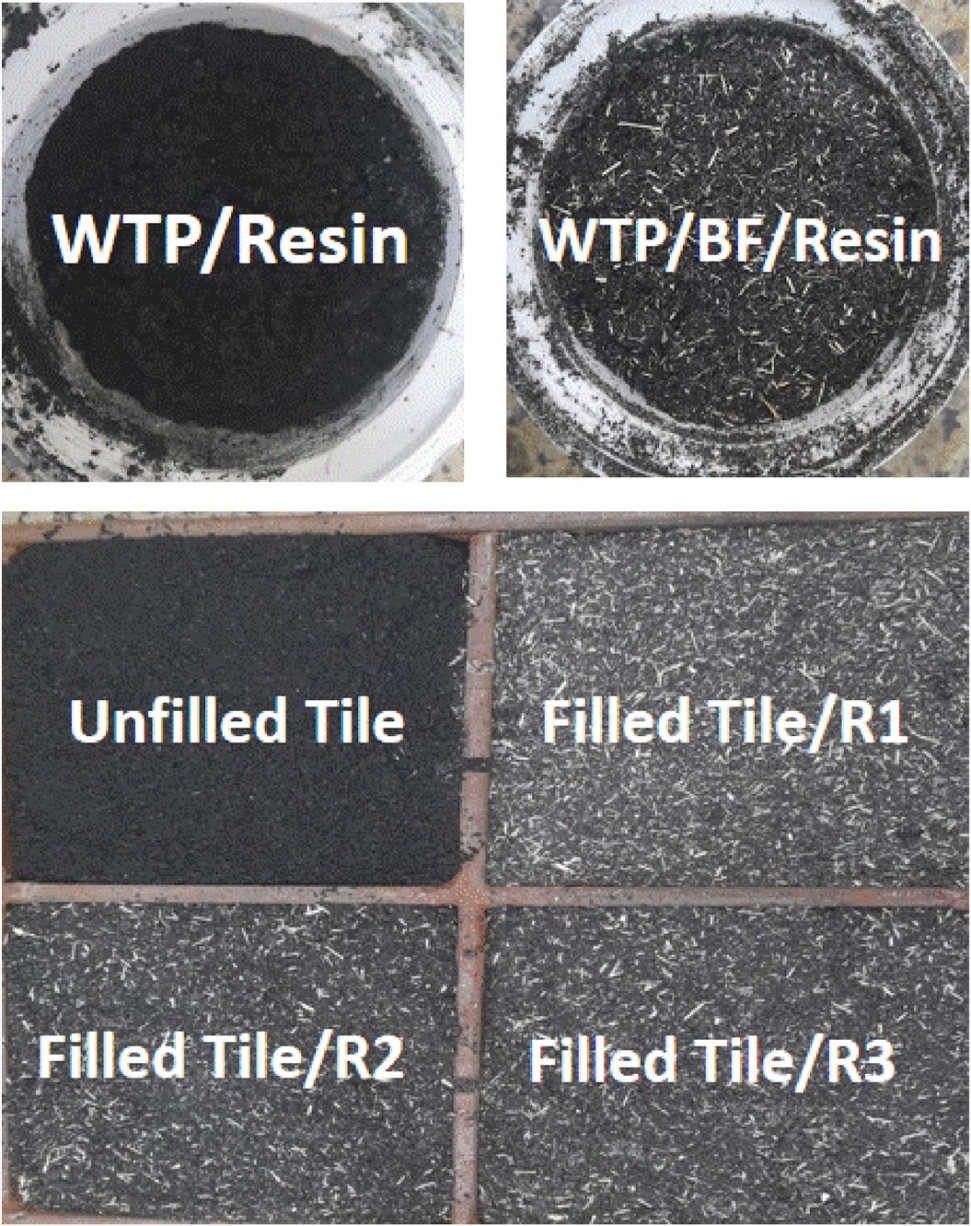
Digital images of unfilled and filled rubber formulations before and after being molded into tiles.

This study aimed to investigate the impact of BF as filler on the physico-mechanical properties of rubber tiles. We were meticulous in our approach, ensuring that the total mixing time was minimized and the temperature was carefully controlled to prevent rubber sticking and cross-linking. Figure 1 provides a schematic representation of the unfilled and filled rubber tiles with resins.

In this study the rationale for reinforcing the waste rubber–epoxy tiles with BF at 0, 5, 10, and 15 wt% is to systematically evaluate how increasing levels of agro-waste filler influences the physico-mechanical and thermal properties of the composites. 0, 5, 10, and 15 wt% formulations represent no bagasse fibers, a low filler loading, and medium loading where fiber–matrix interactions become more pronounced. At high loading content, the tests revealed that the upper limit of reinforcement has been reached before fiber agglomeration and poor dispersion that may be followed by a degrading performance^17,25,26^. A low filler loading (5 wt%) is expected to enhance stiffness and hardness moderately without introducing significant fiber agglomeration. This helps identify the threshold at which mechanical reinforcement begins. A medium loading (10 wt%) is useful to determine whether improvements in compression strength and interfacial adhesion outweigh drawbacks like increased brittleness or abrasion loss. A high loading (15 wt%) contributes in determining the maximum feasible content for balancing strength, hardness, abrasion resistance, and swelling properties.

### Compression or impact resistance of the prepared tiles

**Fig. 2:**
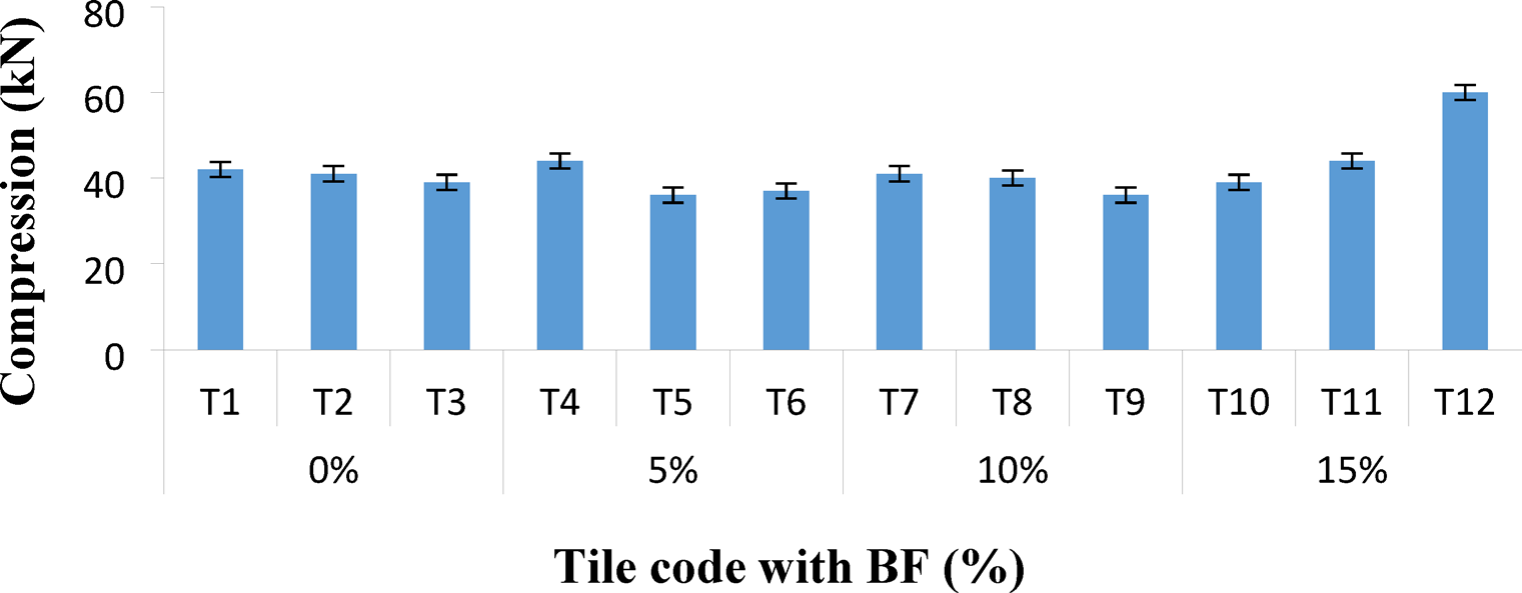
Compression strength of the prepared rubber tiles.

The compression strength values of the prepared tiles were determined as shown in Fig. 2. The prepared rubber tiles were heated and pressed to the optimum cure time. Testing the mechanical properties after adding epoxy to the rubber-bagasse matrix was essential. The study found that the compression strength values of the rubber-bagasse composites reached maxima with a further increase in bagasse contents. Such substantial improvements could be more clearly demonstrated by the arrangement of rubber chains throughout the rubber-bagasse interfacial region after stretching. The enhanced cure properties of the mentioned epoxy resins may be related in some way to their high tensile strength upon elevating the bagasse content.

### Taber rotary abraser

**Fig. 3:**
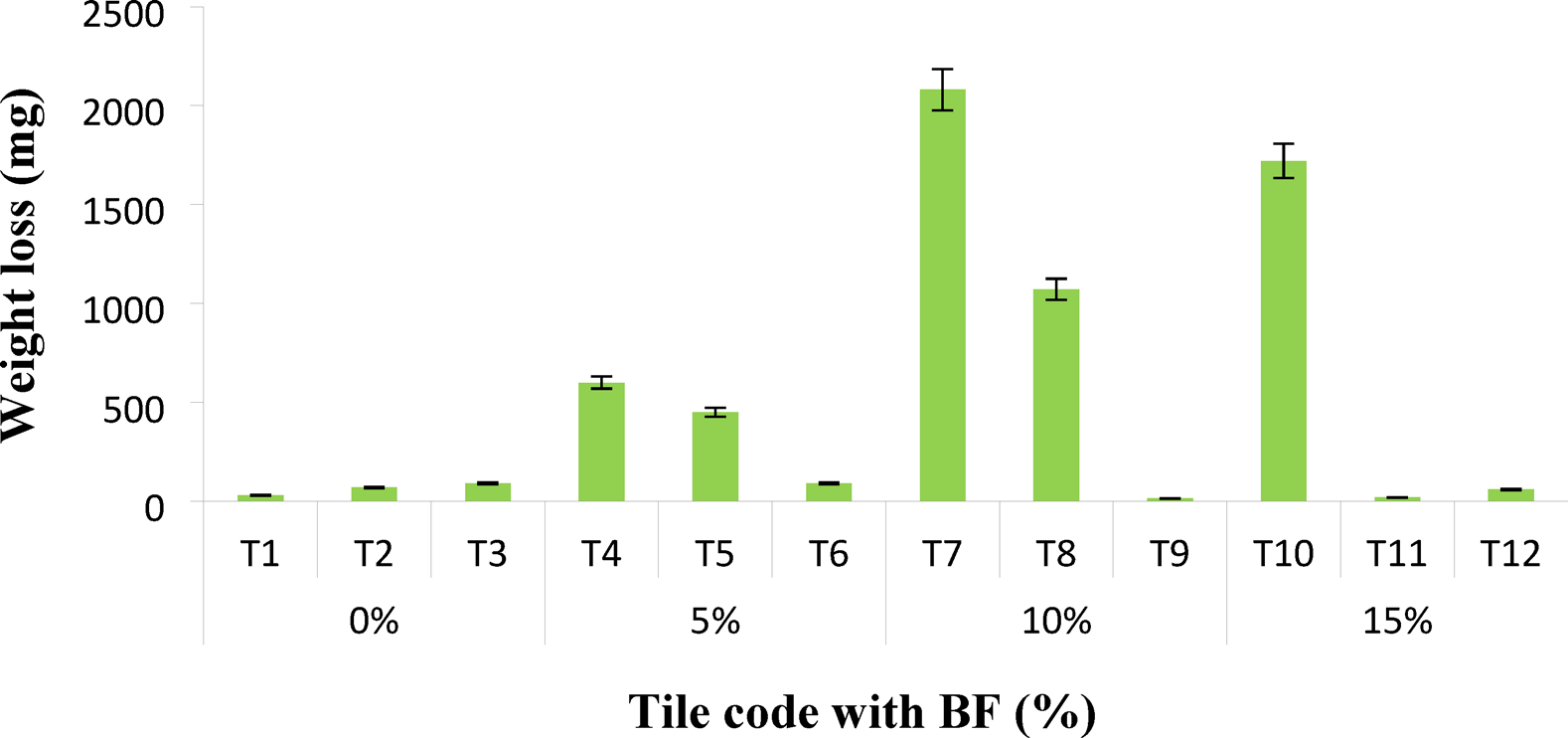
Taber abrasion resistance and wear tests of the prepared rubber tiles.

The abrasion resistance, as indicated by the abrasive weight loss, of the fabricated tiles is depicted in Fig. 3. Notably, at zero bagasse filler, there is no noticeable difference in the weight loss values. However, the abrasive weight loss shows a consistent increase with the rise in bagasse filler. This trend could be attributed to the amplified filler-filler interaction resulting from the bagasse filler’s agglomeration at higher filler loading. The most significant finding is the superior abrasion resistance observed with resin R1 and zero bagasse filler (T1), followed by 10% bagasse with resin R3 (T9) and 15% bagasse with resin R2 (T11). The highest weight loss was recorded with 10% bagasse filler with resin R1 (T7). The enhancement in weight loss with resin R3 in all cases is likely due to the improved filler-rubber interfacial adhesion, leading to enhanced abrasion resistance. These findings underscore the importance of filler-rubber adhesion in improving abrasion resistance.

### Shore hardness

**Fig. 4:**
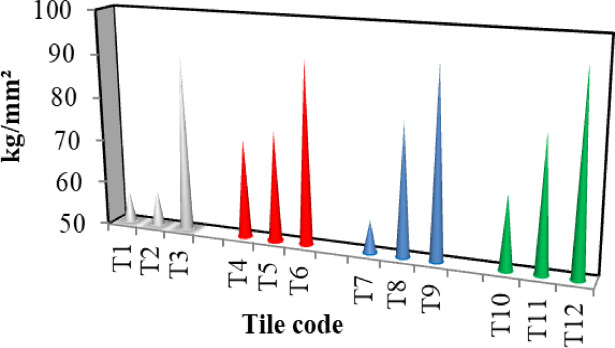
Shore hardness of the prepared tiles.

The ability of a substance to withstand permanent indentation is known as its hardness, the hardness of the different tiles is shown in Fig. 4. The results reveal that the hardness of the prepared tiles increased consistently with increasing bagasse loading. This can be explained as the incorporation of high-rigidity particles, bagasse as filler, reduces the rubber chains’ elasticity, giving high-rigidity rubber tiles^27^. In conclusion, the prepared tiles with R3 show the highest hardness and a plausible explanation is given by the greater reinforcement ability in using this resin.

### Swelling behavior

**Fig. 5:**
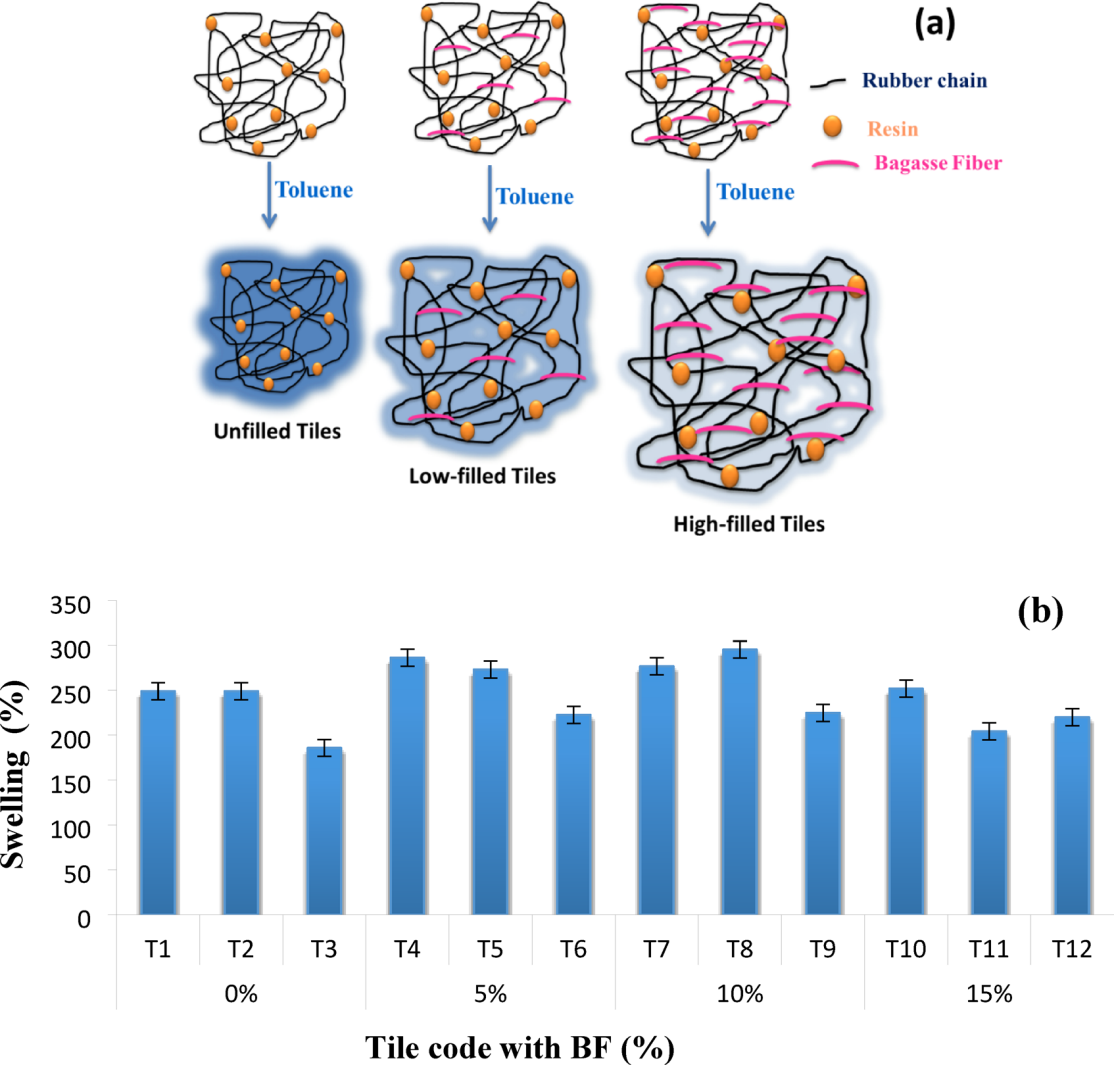
(**a**) Schematic representation for the swelling behavior of unfilled and filled rubber tiles and (**b**) swelling of the prepared rubber tiles in toluene.

The swelling behavior of the rubber–BF tiles showed a clear dependence on the filler content, as shown in Fig. 5. The incorporation of BF led to a noticeable reduction in swelling compared with the unfilled samples, particularly at 5 and 10 wt% loadings. This reduction can be attributed to two main effects: the lignocellulosic composition of BF, rich in cellulose and lignin that promotes strong interfacial interactions with the epoxy–rubber matrix. Thereby, restricting the mobility of polymer chains and limiting solvent uptake arises. Consequently, the physical presence of the rigid fibers reduces the free volume within the composite, guiding to a hindered penetration of toluene molecules. At higher loading (15 wt% BF), partial agglomeration of fibers may generate localized microvoids, which acts as diffusion pathways. Such behavior leads to a slight increase in swelling compared with the 10 wt% sample. These results indicate that moderate BF incorporation enhances the dimensional stability of the composites against solvent attack, while excessive loading may compromise uniform filler dispersion and partially counteract this benefit.

### Morphological study

**Fig. 6:**
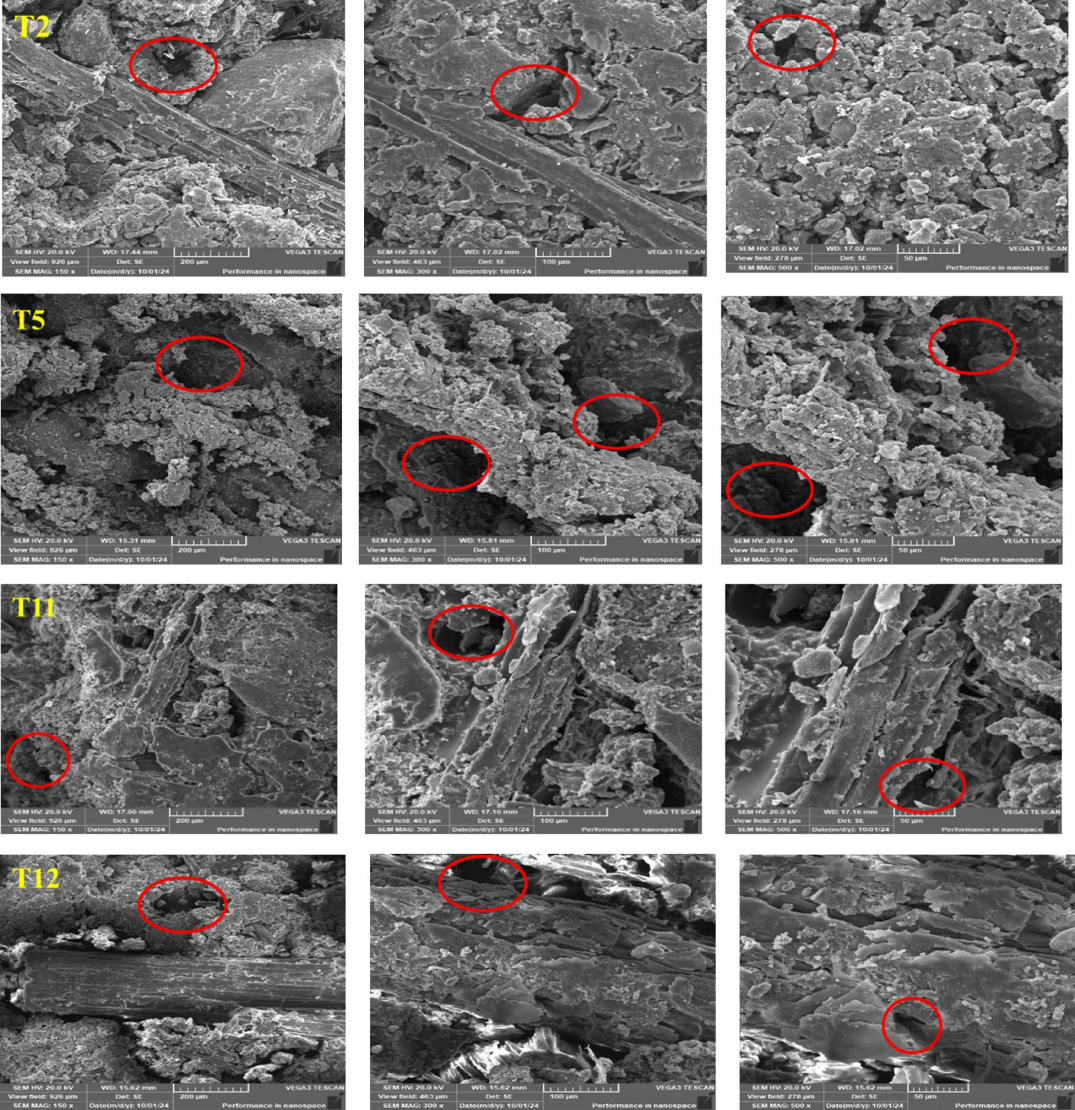
SEM images of T2, T5, T11 and T12 at X150, 300 and 500 magnification.

The surface morphology of the prepared rubber tiles was examined using SEM technique. Representative images of selected samples (T2, T5, T11, and T12) are presented in Fig. 6. These samples were chosen because they represent the formulations with the most notable mechanical responses. At low BF loading (T2), the micrographs show relatively uniform dispersion of waste tire powder with limited fiber–fiber interaction, which corresponds to the moderate compression and hardness values observed. As the bagasse content increases (T5), fiber agglomerates become more pronounced, leading to localized stress concentrations. This morphological feature explains the slight decrease in abrasion resistance compared with lower filler content, since the agglomerates can act as weak points during wear. For samples prepared with resin R2 at 15 wt% bagasse (T11), the SEM images reveal improved interfacial adhesion between the epoxy resin and the rubber–BF matrix. The reduced voids and tighter packing observed in these samples support the high compression strength values reported, confirming that strong resin–BF bonding enhances load transfer and overall mechanical stability. In contrast, the morphology of T12 indicates a greater number of voids and less uniform fiber distribution. These features are consistent with the relatively lower compression and abrasion resistance measured for this formulation. The presence of voids facilitates crack initiation and propagation under load, thereby explaining the weaker performance. Overall, the SEM observations corroborate the mechanical test results: improved filler–resin adhesion and reduced void density. In T11 and R3-based tiles, they are directly associated with higher compression and abrasion resistance, whereas fiber agglomeration or poor dispersion, as in T12, correlates with reduced performance. Thus, the morphological study provides clear microstructural evidence that supports the mechanical behavior of the developed composites.

### Thermogravimetric analysis (TGA)

**Fig. 7:**
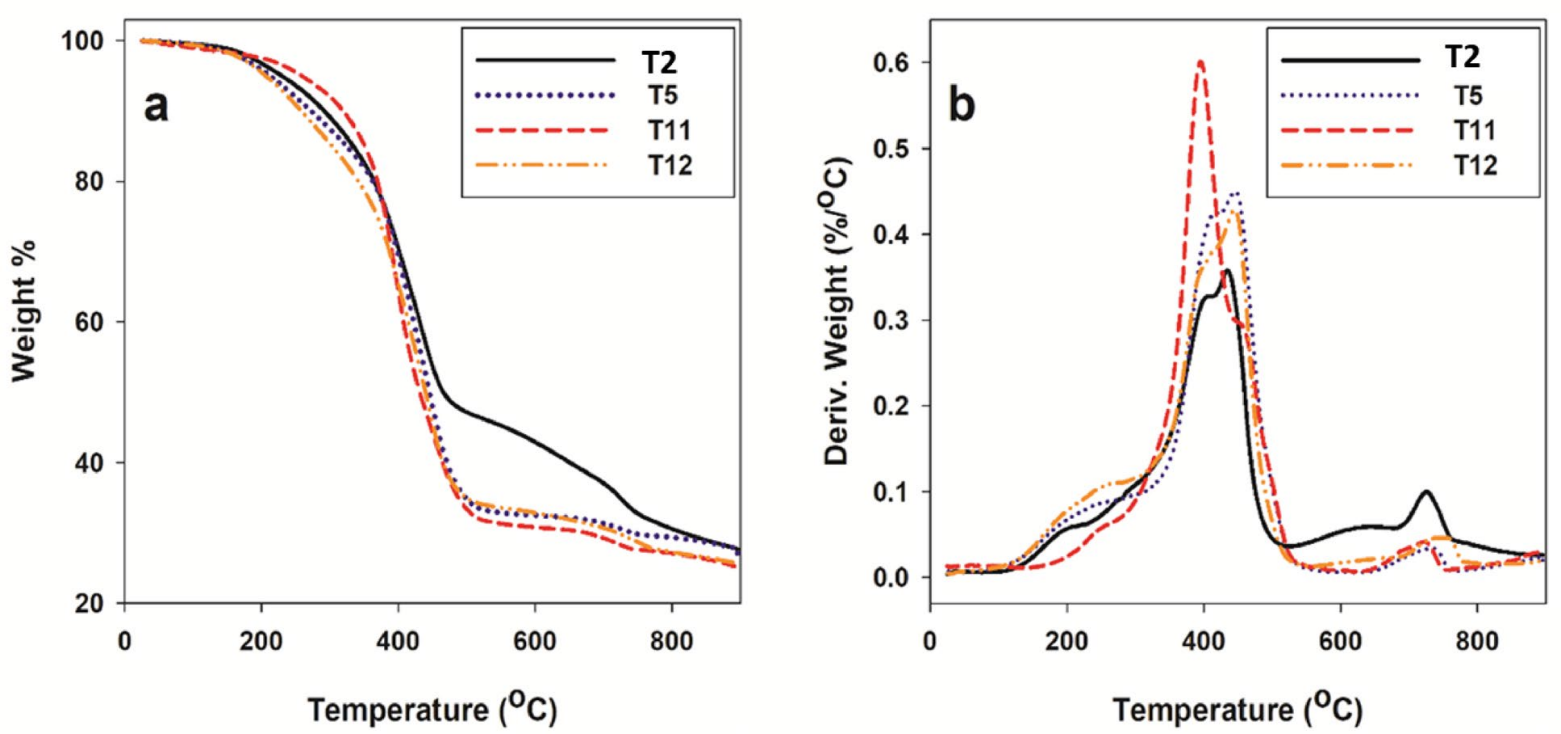
(**a**) TGA and (**b**) DTG curves of T2, T5, T11 and T12 tiles.

TGA has proved to be a suitable method for investigating the thermal stability of prepared rubber tiles. The knowledge of degradation and mode of decomposition under the influence of heat was highly recommended for optimizing process parameters. The decomposition temperature indicates the highest processing temperature that can be used. It was evident that the thermal degradation process for prepared tiles proceeded in five steps. The initial and final degradation temperatures were ∼930 °C. It can be observed that the residue obtained at the degradation amount is 70–75%. Usually, the analysis of TGA data is not straightforward. The rough results cannot be directly converted into absolute features of the studied material. They may depend on factors such as sample mass, heating rate or gas environment. The results reported in this work were specific to the experimental conditions used. The TGA curves of the prepared rubber tiles are shown in Fig. 7a. It was seen from this figure that the weight of the sample decreases continuously as the temperature increases. TGA data relating to the temperatures corresponding to 15% weight loss (T15), 50% weight loss (T50), and maximum weight loss (T_max_) were the main criteria used to indicate the thermal stability of the prepared rubber tiles. The relative thermal stability of the prepared rubber tiles was evaluated by comparing the decomposition temperatures at different weight loss percentages, as shown in Table 3. The higher values of T15, T50, and T_max_ provided higher thermal stability of the investigated systems.

The prepared rubber tiles underwent single-step decomposition over the temperature range from 300 °C to 550 °C. It was also observed that the prepared rubber tiles were stable up to 930 °C. TGA detects the tested samples’ thermal stability and degradation stages. Figure 7b displays the tested samples’ derivative thermogravimetry (DTG) curves at various decomposition steps. T11 shows a maximum decomposition at 397 °C. The initial thermal degradation temperature indicates the thermal stability is noticed for T11, followed by other samples T5 and T12 at higher temperatures till reaching 451 °C. The thermal stability of the analyzed tiles T5, T11 and T12 can be maintained without deterioration compared to the T2 sample. The used epoxy resins contributed to this feature in the prepared tiles.

**Table 3:** TGA decomposition temperatures of prepared rubber tiles.

	Weight loss % at various temperatures (°C)				Decomposition range (°C)	T15	T50
	200	300	400	T_max_			
T2	2	15	25	70	300–550	360	480
T5	4	15	25	70	300–550	320	460
T11	0	12	25	75	300–550	380	430
T12	5	18	25	75	300–550	300	450

## Conclusions

This study demonstrated that bio-based bagasse fibers can be effectively incorporated with waste tire powder in epoxy systems to produce sustainable rubber tiles. The comparative evaluation of three epoxy resins revealed that Kemapoxy 131 (R1) resulted in comparatively lower mechanical and physical performance, while Kemapoxy (R3) produced tiles with superior hardness, swelling resistance, and compression strength at most bagasse loadings. Kemapoxy 175 (R2) offered balanced performance, particularly at 15 wt% bagasse, showing promise for industrial application. Overall, the findings confirm that the choice of epoxy resin plays a decisive role in tailoring the performance of waste-derived rubber tiles. By utilizing low-cost agro- wastes such as bagasse and discarded tire rubber, this approach provides a viable pathway for producing eco-friendly tiles with desirable durability, abrasion resistance, and mechanical strength. These outcomes underline the potential for scaling up this concept into practical manufacturing, thereby contributing to sustainable construction materials and improved waste management strategies.

## Data Availability

All data generated or analyzed during this study are included in this published article.
